# In vitro evaluation suggests fenfluramine and norfenfluramine are unlikely to act as perpetrators of drug interactions

**DOI:** 10.1002/prp2.959

**Published:** 2022-05-22

**Authors:** Parthena Martin, Maciej Czerwiński, Pallavi B. Limaye, Brian W. Ogilvie, Steven Smith, Brooks Boyd

**Affiliations:** ^1^ Zogenix, Inc Emeryville California USA; ^2^ Sekisui XenoTech, LLC Kansas City Kansas USA

**Keywords:** Dravet syndrome, drug transporter, drug–drug interactions, fenfluramine, perpetrator potential

## Abstract

Studies support the safety and efficacy of fenfluramine (FFA) as an antiseizure medication (ASM) in Dravet syndrome, Lennox‐Gastaut syndrome, or CDKL5 deficiency disorder, all pharmacoresistant developmental and epileptic encephalopathies. However, drug–drug interactions with FFA in multi‐ASM regimens have not been fully investigated. We characterized the perpetrator potential of FFA and its active metabolite, norfenfluramine (nFFA), in vitro by assessing cytochrome P450 (CYP450) inhibition in human liver microsomes, CYP450 induction in cultured human hepatocytes, and drug transporter inhibition potential in permeability or cellular uptake assays. Mean plasma unbound fraction was ~50% for both FFA and nFFA, with no apparent concentration dependence. FFA and nFFA were direct in vitro inhibitors of CYP2D6 (IC_50_, 4.7 and 16 µM, respectively) but did not substantially inhibit CYP1A2, CYP2B6, CYP2C8, CYP2C9, CYP2C19, or CYP3A4/5. No time‐ or metabolism‐dependent CYP450 inhibition occurred. FFA and nFFA did not induce CYP1A2; both induced CYP2B6 (up to 2.8‐fold and up to 2.0‐fold, respectively) and CYP3A4 (1.9‐ to 3.0‐fold and 3.6‐ to 4.8‐fold, respectively). Mechanistic static pharmacokinetic models predicted that neither CYP450 inhibition nor induction was likely to be clinically relevant at doses typically used for seizure reduction (ratio of area under curve [AUCR] for inhibition <1.25; AUCR for induction >0.8). Transporters OCT2 and MATE1 were inhibited by FFA (IC_50_, 19.8 and 9.0 μM) and nFFA (IC_50_, 5.2 and 4.6 μM) at concentrations higher than clinically achievable; remaining transporters were not inhibited. Results suggest that FFA and nFFA are unlikely drug–drug interaction perpetrators at clinically relevant doses of FFA (0.2−0.7 mg/kg/day).

## Graphical abstract

Mechanistic static pharmacokinetic models predict that CYP450 inhibition and induction are unlikely for fenfluramine or its major metabolite norfenfluramine at doses used for seizure reduction (0.2−0.7 mg/kg/day). These results suggest minimal drug‐drug interaction perpetrator potential for either fenfluramine or norfenfluramine.

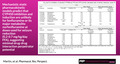

**Abbreviations**
ABC: ATP‐binding cassette transporter
ASM: antiseizure medication
AUC: area under the concentration curve
BCRP: breast cancer resistance protein
BEH: ethylene bridged hybrid
CDD: CDKL5 deficiency disorder
C_max_
: maximum drug concentration
CYP450: cytochrome P450
DDI: drug–drug interaction
DMSO: dimethyl sulfoxide
DS: Dravet syndrome
EC_50_
: concentration of half‐maximal response
EDTA: ethylenediaminetetraacetic acid
E_max_
: maximum induction effect
FDA: United States Food and Drug Administration
FFA: fenfluramine
f_m_
: fraction metabolized
f_u_
: fraction unbound
GAPDH: glyceraldehyde 3‐phosphate dehydrogenase
HEK: human embryonic kidney cell
IC_50_
: potency of inhibition (concentration of 50% inhibition)
I_max__,u_
: maximum unbound systemic plasma concentration of an inhibitor
K_m_
: Michaelis‐Menten rate constant
LC‐MS/MS: liquid chromatography/tandem mass spectrometry
LDH: lactose dehydrogenase
LGS: Lennox‐Gastaut syndrome
LIMS: laboratory information management system
MATE: multidrug and toxin extrusion
MDCKII: Madin‐Darby canine kidney cell
MgCl_2_
: magnesium chloride
mRNA: messenger RNA
NADPH: nicotinamide adenine dinucleotide phosphate
nFFA: norfenfluramine
OAT: organic anion transporter
OCT: organic cation transporter
PBS: phosphate buffered saline
PCR: polymerase chain reaction
P‐gp: P‐glycoprotein multidrug transporter
qRT‐PCR: quantitative reverse transcription‐polymerase chain reaction
R_1_
: the predicted ratio of FFA’s AUC in the presence or absence of an inhibitor
R_3_
: the predicted ratio of FFA’s AUC in the presence or absence of an inducer
RED: rapid equilibration dialysis
S_50_
: substrate concentration at 50% V_max_
SD: standard deviation
SEM: standard error of the mean
SLC: solute carrier transporter
V_max_
: maximum rate of reaction
ΔΔCT: change in threshold cycles

**Significance statement**

Fenfluramine demonstrated efficacy for reducing convulsive seizure frequency in patients with Dravet syndrome and other developmental and epileptic encephalopathies. These patients typically take multiple concurrent antiseizure medications, emphasizing the importance of evaluating fenfluramine’s drug–drug interaction potential. We characterized the perpetrator potential of fenfluramine and its active metabolite, norfenfluramine, in vitro by assessing cytochrome P450 (CYP450) inhibition and induction, as well as drug transporter inhibition potential. Mechanistic static pharmacokinetic models confirm minimal perpetrator potential. A companion paper evaluates fenfluramine’s victim potential.

## 1 INTRODUCTION

Fenfluramine (FFA), as designated by International Union of Pharmacology (IUPHAR),^1^ or (RS)‐ethyl(α‐methyl‐3‐trifluoromethylphenethyl)amine, has shown efficacy in clinical trials as an adjunctive antiseizure medication (ASM) for treatment of patients with Dravet syndrome (DS), Lennox‐Gastaut syndrome (LGS), and CDKL5 deficiency disorder (CDD). All are rare developmental and epileptic encephalopathies characterized by frequent pharmacoresistant seizures.^2^, ^3^ FFA was approved for treatment of Dravet syndrome in the US, EU, and UK, and was recently approved for Lennox‐Gastaut syndrome in the US. Pharmacoresistance in these patients often results in polypharmacy to reduce seizure frequency; most patients are prescribed three to four concurrent ASMs.^4^, ^5^, ^6^, ^7^

During the FFA development program, the potential for drug–drug interactions (DDIs) when FFA is added to existing ASM regimens was evaluated in vivo. Initial treatment for patients with LGS is usually valproate, lamotrigine, and/or topiramate, followed by adjunctive felbamate, clobazam, levetiracetam, and cannabidiol.^5^, ^8^ Patients with DS typically are first prescribed valproate and clobazam, with refractory seizures treated by adding stiripentol.^9^ Adjunctive cannabidiol and FFA are more recently developed treatment options^2^, ^3^, ^5^; clonazepam, levetiracetam, and zonisamide, with ethosuximide for atypical absence seizures,^9^ are additional options. CDD does not currently have a targeted therapy, although recent open‐label trials support clinical efficacy of both cannabidiol and FFA in reducing median convulsive seizure frequency.^10^, ^11^

FFA undergoes de‐alkylation to norfenfluramine (nFFA)^12^, ^13^ in the liver by the cytochrome P450 (CYP450) mixed‐function oxidase system. Therefore, both FFA and nFFA were evaluated for their drug interaction potential. CYP1A2, CYP2B6, CYP2C8, CYP2C9, CYP2C19, CYP2D6, CYP3A4, and CYP3A5 catalyze most drug biotransformation reactions. These enzymes are commonly implicated in DDIs.^14^, ^15^ The pharmacokinetics of ASMs used to treat LGS and/or DS, such as clobazam and cannabidiol, may have victim potential when used in combination with moderate or strong CYP450 inhibitors and may require dose adjustments due to DDIs.^16^, ^17^

Polypharmacy is typically used to manage the multiplicity of seizures characteristic of developmental and epileptic encephalopathies. FFA will be used in combination regimens, necessitating a thorough characterization of potential for DDIs. The FDA and EMA have developed comprehensive “Guidance for Industry” documents which outline the most current state of the science for predicting DDIs. These documents use “a systematic, risk‐based approach to assessing DDI potential of investigational drugs and making recommendations to mitigate DDIs.”^15^ The recommended assays provide a robust, rigorous, systematic investigation into both victim and perpetrator potential of an investigational drug. The outcome of these experiments is aimed to provide regulatory support for the investigational product and inform clinical practice when prescribing FFA in combination ASM regimens.

Perpetrator (or precipitant) ASMs may affect the clearance, efficacy, and/or toxicity of victim (or object) ASMs in combination therapy, especially if the perpetrator induces or inhibits an enzyme responsible for a single‐elimination pathway.^18^, ^19^, ^20^ Perpetrator ASMs can also inhibit drug transporter proteins widely distributed throughout the body, thereby modulating drug pharmacokinetics and drug action via modifications in absorption, distribution, tissue‐specific drug targeting, and elimination of the victim drug.^21^ US FDA guidance for industry recommends that sponsors conduct a comprehensive panel of in vitro metabolism‐ and transporter‐mediated DDI studies as part of their preclinical development programs.^15^

In this study, we performed in vitro DDI studies in accordance with FDA guidance to identify the CYP450 enzymes and transporter proteins that could be altered and contribute to the perpetrator potential of FFA and its major metabolite, nFFA, in the context of multi‐ASM dosing regimens.^15^, ^22^ A companion paper characterizes the victim potential of FFA and nFFA.^23^

## 2 MATERIALS AND METHODS

### 2.1 Chemicals

The CYP450 substrates phenacetin, diclofenac, dextromethorphan, midazolam, and testosterone were obtained from Sigma‐Aldrich (St. Louis, MO); efavirenz and amodiaquine from the US Pharmacopeia (Rockville, MD); and *S*‐mephenytoin from Toronto Research Chemicals (Toronto, Ontario, Canada). Substrate metabolites 4′‐hydroxy‐diclofenac and 6β‐hydroxytestosterone were obtained from Sigma‐Aldrich; acetaminophen, *N*‐desethylamodiaquine, 4′‐hydroxymephenytoin, dextrorphan, and 1′‐hydroxymidazolam from Cerilliant (Round Rock, TX); and 8‐hydroxyefavirenz from Toronto Research Chemicals. Internal standards for all CYP450 bioanalytical assays were as follows: acetaminophen‐d_4_ (Cerilliant Corporation), 8‐hydroxyefavirenz‐d_4_ (Toronto Research Chemicals), *N*‐desethyl‐amodiaquine‐d_5_ (Toronto Research Chemicals), 4′‐hydroxydiclofenac‐d_4_ (TLC Pharmaceutical Standards, Aurora, Ontario, Canada), 4′‐hydroxymephenytoin‐d_3_ (TLC Pharmaceutical Standards), dextrorphan‐d_3_ (Toronto Research Chemicals), 1′‐hydroxymidazolam‐d_4_ (Cerilliant Corporation), and 6β‐hydroxy‐testosterone‐d_3_ (Cerilliant Corporation). The α‐naphthoflavone, orphenadrine, modafinil, quinidine, and ketoconazole used as positive controls for direct inhibition of CYP450s were obtained from Sigma‐Aldrich; montelukast from Sequoia Research Products (Pangbourne, United Kingdom); and sulfaphenazole from Santa Cruz Biotechnology (Dallas, TX). Tienilic acid (Cypex, Ltd., Dundee, Scotland), esomeprazole (Toronto Research Chemicals), furafylline, phencyclidine, paroxetine (Sigma‐Aldrich), troleandomycin (Enzo Life Sciences, Farmingdale, NY), and gemfibrozil glucuronide (XenoTech, Kansas City, KS) were used as positive controls for metabolism‐dependent CYP450 inhibition. Components of the NADPH‐generating system (β‐NADP, glucose‐6‐phosphate, glucose‐6‐phosphate dehydrogenase) were obtained from Sigma‐Aldrich. FFA hydrochloride and nFFA hydrochloride—[1‐(3‐trifluoromethyl) phenyl]propan‐2‐amine hydrochloride—were obtained from Zogenix, Inc. (Emeryville, CA). Flumazenil, omeprazole, phenobarbital, and rifampin used in CYP450 induction studies were obtained from Sigma‐Aldrich.

For studies examining cell membrane transporters, the following agents were all obtained from Sigma‐Aldrich: digoxin, valspodar, verapamil, prazosin, Ko143, estradiol‐17β‐glucuronide, rifampin, cyclosporine, butyric acid, *p*‐aminohippuric acid, probenecid, novobiocin, estrone‐3‐sulfate, ibuprofen, and quinidine. Digoxin‐d_3_ and prazosin‐d_8_ were obtained from Toronto Research Chemicals; lopinavir from the European Pharmacopoeia (Strasbourg, France); [^3^H]‐*p*‐aminohippuric acid from American Radiolabeled Chemicals (St. Louis, MO); [^3^H]‐estradiol‐17β‐glucuronide and [^3^H]‐estrone‐3‐sulfate from PerkinElmer (Hopkinton, MA, USA); and [^14^C]‐metformin from Moravek, Inc. (Brea, CA).

### 2.2 Direct CYP450 inhibition

Inhibition of CYP450s by FFA and nFFA was assessed according to methods described previously.^19^, ^24^, ^25^ Microsomes from non‐transplantable human livers were prepared and characterized as outlined previously.^25^, ^26^ A mixed‐gender pool of 200 individual human liver microsomal samples was used. Substrate concentrations and incubation conditions for determination of the kinetic constant (S_50_ or K_m_) used to select marker substrate concentrations were previously determined.^24^ FFA and nFFA were each evaluated for ability to inhibit the following CYP450‐selective reactions in a direct, time‐dependent, and metabolism‐dependent manner: CYP1A2, phenacetin O‐dealkylation; CYP2B6, efavirenz 8‐hydroxylation; CYP2C8, amodiaquine N‐dealkylation; CYP2C9, diclofenac 4′‐hydroxylation; CYP2C19, *S*‐mephenytoin 4′‐hydroxylation; CYP2D6, dextromethorphan O‐demethylation; CYP3A4/5, midazolam 1′‐hydroxylation; and CYP3A4/5, testosterone 6β‐hydroxylation.

Aliquots of FFA or nFFA solutions were added to buffer (pH 7.4) containing potassium phosphate buffer (50 mM), MgCl_2_ (3 mM), and EDTA (1 mM). Incubations to measure CYP450 enzyme activity were conducted at approximately 37°C in 200‐µL incubation mixtures containing the buffer mixture described previously, an NADPH‐generating system (mixture of the following: NADP [1 mM], glucose‐6‐phosphate [5 mM], glucose‐6‐phosphate dehydrogenase [1 Unit/mL]), and a concentration of marker substrate based on the K_m_ or S_50_ determined previously. Concentrations of FFA ranged from 0.06−60 µM for all CYP450 enzymes examined except for CYP3A4/5. CYP3A4/5 inhibition was measured using higher concentrations of FFA ranging from 0.6−600 µM with the intention of identifying the potential of this test article to cause interactions with CYP3A4/5 in the intestine after oral administration. Concentrations of nFFA ranged from 0.1−100 µM. Detailed analytical and statistical methods for determining the degree of inhibition can be found in the Supplemental Material.

### 2.3 Metabolism‐dependent CYP450 inhibition

To examine metabolism‐dependent inhibition of the CYP450 enzymes, FFA and nFFA were preincubated in duplicate at 37 ± 1°C with human liver microsomes as described above for ~30 min in the presence of an NADPH‐generating system, which allowed for the generation of potential intermediates that could irreversibly inhibit human CYP450 enzymes. For comparison, additional duplicate samples containing FFA or nFFA were preincubated for 30 min in the absence of NADPH. These preincubations allowed assessment of the NADPH‐dependence of any time‐dependent CYP450 inhibition. Following the 30‐min preincubation, the NADPH‐generating system and/or marker substrate was added, and incubations were continued as described previously to measure residual CYP450 enzyme activity. Incubations that contained FFA and nFFA but were not preincubated served as negative controls for time‐dependent CYP450 inhibition.

### 2.4 Red blood cell/plasma partition

To determine the extent of red blood cell accumulation of FFA and nFFA, the red blood cell/plasma partition of both drugs was assessed in human blood obtained from BioIVT (Westbury, NY). Fresh reference plasma was separated from whole blood by centrifugation at 2000 × *g* (5 min, 4°C). FFA or nFFA (2 µM in dimethyl sulfoxide [DMSO]) or the reference assay control (chloroquine) was incubated in both whole blood and reference plasma (60 min, 37°C; final DMSO concentration, 0.1%) and was centrifuged (2000 × *g*; 5 min, 4°C). Plasma (30 µL) was aliquoted to multiwall plates and was quenched with 300 µL of 50% acetonitrile, 50% methanol, and 0.05% formic acid solution containing internal standards (bucetin and warfarin). Plates were vortexed and centrifuged (4000 RPM, 15 min, 4°C); supernatants were transferred to fresh plates for quantification by liquid chromatography/tandem mass spectrometry (LC‐MS/MS; API 4000 [AB Sciex, Framingham, MA] coupled to a Shimadzu LC‐20AD LC pump system [Shimadzu Scientific Instruments, Columbia, MD] using Atlantis T3 dC18 reverse phase HPLC columns [Waters Corporation, Milford, MA]; 20 mm × 2.1 mm; flow rate: 0.5 mL/min; 3‐min gradient starting and ending with 98% solvent A, where the mobile phase consisted of solvent A—0.1% formic acid in water, and solvent B—0.1% formic acid in acetonitrile). Red blood cell‐to‐plasma ratio was calculated as follows:

KRBC/PL=1/H(Cp/Cb‐1)+1,

where:

H = hematocrit (percent of total blood cells in whole blood sample, v/v),
Cp = ratio of peak areas of the analyte over internal standard in reference plasma,
Cb = ratio of peak areas of the analyte over internal standard in top plasma layer separated from whole blood (i.e., concentration in the plasma layer after centrifugation of blood samples, not concentration in the blood).

### 2.5 CYP450 induction

Measurement of CYP450 induction in human hepatocyte cultures was performed as previously described.^27^ Briefly, cultures were obtained from three lots of cryopreserved hepatocytes (Sekisui XenoTech, Kansas City, KS) and were treated once daily for 3 consecutive days with 0.1% v/v DMSO (vehicle), flumazenil (25 μM, negative control), one of six concentrations of FFA (0.5, 1, 4, 10, 20, or 40 μM), one of six concentrations of nFFA (0.5, 1, 5, 10, 25, or 50 μM), or one of three positive control CYP450 enzyme inducers, namely, omeprazole (50 μM), phenobarbital (750 μM), or rifampin (20 μM). Approximately 24 h following final treatment, cultures were visualized with a Nikon TMS Microscope (Nikon Corporation, Tokyo, Japan) or an Accu‐Scope 3020 Inverted Microscope (Accu‐Scope Inc., Commack, NY), and a representative dish from each treatment group was photographed with a PAXcam5 digital camera (MIS Inc., Huntingdon Valley, PA) to document morphological integrity. Cells were then harvested to isolate RNA, which was analyzed by quantitative reverse transcription‐polymerase chain reaction (qRT‐PCR) for induction of CYP1A2, CYP2B6, and CYP3A4 mRNA levels. Total RNA was phase extracted with TRIzol followed by purification with an RNeasy Mini Kit (Qiagen Inc., Germantown, MD). Quantitative PCR was performed in triplicate with a 7900HT Fast Real Time PCR System, an Applied Biosystems Universal Master Mix, and TaqMan^®^ Gene Expression Assays. The quantity of target cDNA relative to control cDNA (GAPDH) was determined by the ΔΔCT method (Applied Biosystems User Bulletin #2). Relative quantitation measures the change in mRNA expression in a test sample relative to that in the control sample (i.e., DMSO). This method assumes that the efficiency of target amplification and the efficiency of endogenous control amplification are approximately equal.

The potential of FFA and nFFA to cause cytotoxicity was assessed based on the release of lactate dehydrogenase (LDH) into the culture medium (Sigma‐Aldrich Cytotoxicity Detection Kit [LDH] Cat. # 11644793001) and based on daily microscopic evaluation.

### 2.6 Protein binding

FFA and nFFA were each added to triplicate samples of pooled rat, dog, and human plasma (final concentrations, 10 and 100 ng/mL). In vitro plasma protein binding was then determined by using the Rapid Equilibrium Device (RED) with a molecular weight cutoff of 8 kDa (Thermo Fisher Scientific, Inc., Waltham, MA). The warfarin positive control was added to separate samples of pooled human plasma for a final concentration of 10 μM. The internal standard was niflumic acid. FFA and nFFA (1 and 10 ng/mL each) were added to plasma of each species, mixed gently, and stored in an ice bath until subsequent analysis. Protein binding was determined by adding a 300‐μL aliquot of the test sample or the control sample to the sample chamber of the RED and adding a 500‐μL aliquot of PBS (pH 7.4) to the buffer chamber. The device unit was sealed and incubated at 37°C for 4 h with gentle shaking (~750 rpm). After dialysis, three aliquots of the test samples (50 μL) were collected from both sample and buffer chambers and were mixed with 50 μL of methanol as “donor samples” and “acceptor samples,” respectively. Initial plasma samples and donor samples, acceptor samples, samples for calibration curves, and samples for positive control were spiked with the internal standard and were subjected to LC‐MS/MS quantification of FFA and nFFA. Concentrations for test samples were determined by regression from peak area values obtained from standard curves (FFA, 0, 1, 3, 10, 30, 100, and 300 ng/mL; nFFA, 0.1, 0.3, 1, 3, 10, 30, and 100 ng/mL). A detailed description of the LC‐MS/MS process can be found in the Supplemental Material. Supernatant fractions were analyzed by LC‐MS/MS using an Acquity Ultra Performance Liquid Chromatography system with ethylene bridged hybrid (BEH) C18 columns (2.1 × 50 mm, 1.7 µm; Waters Corporation; Milford, MA) coupled to an API4000 mass spectrometer (AB Sciex). Calibration curves were determined by dissolving FFA or nFFA in methanol to prepare curves of 0, 1, 3, 10, 30, 100, and 300 ng/mL for FFA, and 0, 0.1, 0.3, 1, 3, 10, 30, and 10 ng/mL for nFFA. Samples were prepared in triplicate and were dialyzed for 4 h. Peak area values were obtained from LC‐MS/MS chromatograms corresponding to the analyte and the internal standard. Analyst software (v. 1.4.2; AB Sciex) was used to calculate a regression equation based on the calibration curve by weighted least squares and concentrations of analyte in the samples. Protein binding was calculated as follows:

$$\begin{array}{c} \text{Protein}\;\text{binding}\;\text{ratio}\;\left(\%\right)=100‐\text{Unbound}\;\text{ratio}\;\left(\%\right), \end{array}$$

where

$$\begin{array}{c} \text{Unbound}\;\text{ratio}\;\left(\%\right)=\frac{\text{Acceptor}\;\text{Sample}\;\text{Concentration}}{\text{Donor}\;\text{Sample}\;\text{Concentration}}. \end{array}$$

### 2.7 Transporter inhibition

FFA and nFFA were evaluated as inhibitors of the human ATP‐Binding Cassette Transporters (ABC) BCRP and P‐gp and the Solute Carrier (SLC) transporters OAT1, OAT3, OCT2, MATE1 and MATE2‐K, OATP1B1, and OATP1B3, according to published methods (Supplemental Table 1).^28^ FFA and nFFA were evaluated as inhibitors of the P‐gp transporter in Caco‐2 cells (American Type Culture Collection, Manassas, VA) and of the BCRP transporter in MDCKII‐BCRP cells (Netherlands Cancer Institute, Amsterdam, Netherlands). For remaining transporters, HEK293 cells transfected with transporter‐specific viral vectors (Sekisui Medical Co. Ltd., Tokyo, Japan) were used to assess FFA and nFFA inhibitory effects as described.^29^ The test compound (FFA or nFFA) or the positive control substrate (digoxin and prazosin for BCRP and P‐gp transporters, respectively) was added to the donor chamber. Samples were collected from the receiver compartment at 15, 30, and 120 min, mixed with an internal standard, and analyzed for FFA, nFFA, or control substrate by LC‐MS/MS. For uptake transporter inhibition studies, transporter‐transfected cells and control cells were preincubated in medium containing FFA, nFFA, positive control, or solvent control for 15 min, followed by a second incubation in the presence of a radiolabeled probe substrate for 1 to 2 min. Cells were then washed, lysed with scintillation cocktail, and analyzed for the probe substrate with a MicroBeta scintillation counter (PerkinElmer, Hopkinton, MA). Transcellular transporter assay conditions are described in Supplemental Table 1. Calculation of IC_50_ values was based on bidirectional permeability of probe substrate with and without the inhibitor in MDCKII cells expressing BCRP and in Caco‐2 cells expressing P‐gp, and transporter‐dependent uptake of probe substrate in HEK293 cells expressing all other transporters (Supplemental Table 1).

### 2.8 Statistical analysis and data analysis

Summary data are expressed as percent of control and mean ± standard deviation (SD) or standard error of the mean (SEM), as appropriate. Individual values from inhibition studies were processed with the laboratory information management system (LIMS) Galileo v3.3 (Thermo Fisher Scientific), which was used to calculate IC_50_ values from a four‐parameter logistic regression applied to percent inhibition versus concentration values. For induction data, IC_50_ and E_max_ were obtained by applying the fitting algorithm in SigmaPlot 12.5 to fold‐increase and concentration values to obtain the best‐fit three‐parameter logistic function. Based on FDA guidance for metabolism‐ and transporter‐mediated DDI studies,^15^ basic kinetic models for transporter inhibition, CYP450 inhibition, and CYP450 induction were employed to calculate R, R_1_, and R_3_ values, respectively, and were compared to the respective predetermined threshold values listed in the guidance document.^15^ Mechanistic static models for CYP450 inhibition and induction were employed to calculate AUCR and were compared to threshold values (AUCR >1.25 to denote inhibition and AUCR <0.8 to denote induction).^15^ The model assumed a maximum daily dose of 26 mg/day FFA base administered in a twice‐daily dose of 13 mg and 100% metabolism by the target CYP450s (Supplemental Table 2). In clinical studies, FFA was administered twice daily as FFA HCl oral solution (2.2 mg/mL FFA base).^2^, ^3^ Plasma protein binding data were used to estimate input parameters for fraction unbound to plasma proteins in the mechanistic static model (Supplemental Table 2). The fraction absorbed was assumed to be 100%, yielding the highest possible portal concentrations. The k_a_ was assumed to be 0.69/h for FFA. Based on population pharmacokinetic studies, maximum plasma concentrations of 68 and 37.8 ng/mL were used as model parameters for nFFA and FFA, respectively (Prescribing information: https://www.fintepla.com/). Assays were performed in duplicate or triplicate according to previously published reports. Statistical analysis for replicates was not possible with *n *= 2 sample sizes.

### 2.9 Nomenclature of targets and ligands

Key protein targets and ligands in this article are hyperlinked to corresponding entries in http://www.guidetopharmacology.org, the common portal for data from the IUPHAR/BPS Guide to PHARMACOLOGY,^30^ and are permanently archived in the Concise Guide to PHARMACOLOGY 2021/22^31^, ^32^, ^33^ and the IUPHAR/BPS Guide to Pharmacology Database.^1^

## 3 RESULTS

### 3.1 Plasma protein binding

Protein binding of the positive control, 10 µM warfarin, was 99.5 ± 0.2% in human plasma, in close agreement with published data.^3^, ^34^ Mean plasma unbound fraction FFA at 10 or 100 ng/mL in human plasma was 55.2 ± 7.8% and 49.9 ± 1.8%, respectively. Mean nFFA unbound fraction at 10 or 100 ng/mL was 50.3 ± 12.7% and 52.0 ± 2.0%, respectively. The unbound fraction of FFA and nFFA in rat or dog plasma was in the range of 37.8 ± 8.6% to 53.8 ± 2.2% and 44.5 ± 7.2% to 56.2 ± 6.8%, respectively. Recovery of FFA and nFFA ranged from 72.2 ± 1.4% to 115.9 ± 11.1% (all species). Thus, plasma protein binding was approximately 50% for both FFA and nFFA in human plasma.

### 3.2 CYP450 inhibition

**FIGURE 1:**
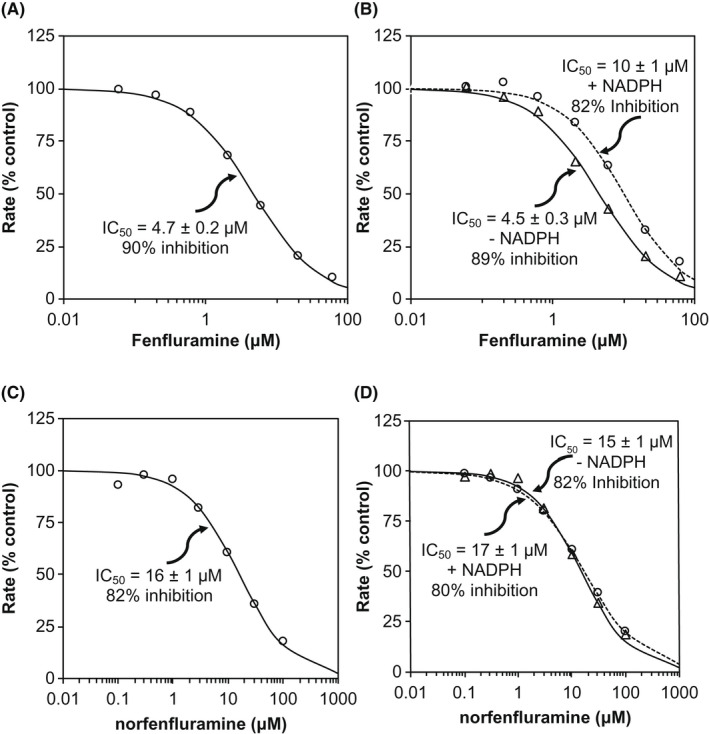
Direct, but not time‐ or metabolism‐dependent, inhibition of CYP2D6 (dextromethorphan O‐demethylation) in human liver microsomes by fenfluramine (A, B) or norfenfluramine (C, D). Assays were conducted (A, C) without a 30‐min preincubation or (B, D) with a 30‐min preincubation in the presence (open circles) or absence (open triangles) of NADPH

The inhibitory effects of FFA and nFFA on CYP450 enzymes in terms of IC_50_, percentage of inhibition, R_1_ values calculated for the basic model of reversible inhibition, and AUCR values calculated for the static mechanistic model are shown in Table 1. The calculated R_1_ value is the ratio of intrinsic clearance values of a probe substrate for an enzymatic pathway in the absence and in the presence of FFA or nFFA.^15^ Static mechanistic models incorporate more detailed drug disposition and drug interaction mechanisms for both interacting and substrate drugs. Under experimental conditions measuring direct enzyme inhibition, all FFA IC_50_ values for CYP1A2, CYP2B6, CYP2C8, CYP2C9, and CYP2C19 were above 60 µM—the highest concentration of the drug tested. FFA inhibited these enzymes by 2.4%–14%. FFA directly inhibited 90% of CYP2D6 activity with an IC_50_ of 4.7 ± 0.2 μM (Figure 1A). FFA IC_50_ values for CYP3A4/5 were above 600 µM, the highest concentration of drug tested, for both of the CYP3A4/5 substrates, namely, midazolam and testosterone. FFA inhibited up to 18% of CYP3A4/5 testosterone 6β hydroxylase activity. All nFFA IC_50_ values for CYP1A2, CYP2B6, CYP2C8, CYP2C9, and CYP2C19 were above 100 µM—the highest concentration tested. nFFA inhibited these enzymes by 2.9%–23%. nFFA directly inhibited 82% of CYP2D6 activity, with IC_50_ of 16 ± 1 μM (Figure 1C). nFFA inhibited up to 2.8% of CYP3A4/5 testosterone 6β hydroxylase activity. Under experimental conditions to measure time‐ or metabolism‐dependent inhibition, FFA and nFFA did not have any additional inhibitory effects on CYP450 enzyme activity compared to direct inhibition (Figure 1B, D for CYP2D6).

**TABLE 1:** IC_50_ and % of inhibition of selected CYP450s by Fenfluramine (FFA) and norfenfluramine (nFFA) and the calculated ratio of intrinsic clearance values R_1_

CYP	Substrate	IC_50_ (µM)	Inhibition (%)	Basic model		Static mechanistic model	
Enzyme				R_1_	Potential for clinical inhibition^a^	AUCR	Potential for clinical inhibition^c^
FFA							
CYP1A2	Phenacetin	>60	NC	NC	No	1.01	No
CYP2B6	Efavirenz	>60	6.1	1.01	No	1.01	No
CYP2C8	Amodiaquine	>60	5.9	1.01	No	1.01	No
CYP2C9	Diclofenac	>60	2.4	1.01	No	1.01	No
CYP2C19	*S*‐Mephenytoin	>60	14	1.01	No	1.01	No
CYP2D6	Dextromethorphan	4.7 ± 0.2	90	1.07	Yes	1.17	No
CYP3A4	Midazolam	>600	NC	NC R_1,gut_ = 1.78	No No^b^	1.00	No
CYP3A4	Testosterone	>600	18	1.00 R_1,gut_ = 1.81	No No^b^	1.00	No
nFFA							
CYP1A2	Phenacetin	>100	NC	NC	No	1.00	No
CYP2B6	Efavirenz	>100	23	1.00	No	1.00	No
CYP2C8	Amodiaquine	>100	NC	NC	No	1.00	No
CYP2C9	Diclofenac	>100	NC	NC	No	1.00	No
CYP2C19	*S*‐Mephenytoin	>100	2.9	1.00	No	1.00	No
CYP2D6	Dextromethorphan	16 ± 1	82	1.01	No	1.01	No
CYP3A4	Midazolam	>100	NC	NC	No	1.00	No
CYP3A4	Testosterone	>100	2.8	1.00	No	1.00	No

Note

NC, not calculated. No value was obtained, as rates of metabolite formation were higher than control rates.

R_1_ = 1 + (I_max,u_/K_i,u_); R_1,gut_ = 1 + (I_gut_/K_i,u_) and [I]_gut_ = dose (µmol)/0.25 L (U.S. FDA, 2020).

AUCR = (1/[A_g_ × B_g_] × (1−F_g_) + F_g_) × (1/[A_h_ × B_h_] × f_m_ + (1 − f_m_)).

^a^ Potential to inhibit if R_1_ ≥ 1.02.

^b^ Potential to inhibit if R_1,gut_ ≥ 11.

^c^ Potential to inhibit if AUCR ≥ 1.25.

FFA and nFFA potential to inhibit CYP450 enzymes in the clinic was evaluated initially with the basic model and then with the static mechanistic model.^15^ Calculated FFA or nFFA R_1_ values for CYP1A2, CYP2B6, CYP2C8, CYP2C9, CYP2C19, and CYP3A4/5 were below the threshold level of 1.02, indicating that FFA and nFFA had only minimal potential to inhibit these enzymes in vivo (Table 1).

FFA and nFFA R_1_ values for CYP2D6 (1.07 and 1.01, respectively. Since the FFA value was greater than the basic model threshold value and indicated its potential to inhibit the enzyme in the clinic, the potential of FFA and nFFA to inhibit CYP2D6 was investigated further with the static mechanistic model.^15^ In this model, the predicted AUCR of an index substrate in the presence and in the absence of FFA equaled 1.17, and was lower than the threshold value of 1.25, indicating that a clinical study of FFA inhibition potential for CYP2D6 is unlikely to be necessary. The nFFA CYP2D6 AUCR value was 1.01, indicating that a clinical study of nFFA inhibition potential was also unlikely to be necessary.

The FFA potential to inhibit intestinal CYP3A4/5 was evaluated with the basic model. The calculated R_1,gut_ value was <1.8 and was lower than the threshold value of 11, indicating that clinically relevant inhibition of CYP3A4/5 in the gut by FFA was unlikely.

### 3.3 CYP induction

At the time of isolation for cryopreservation, the viability of hepatocyte preparations used for induction assays was between 81.7% and 91% (Supplemental Table 3). Cells treated with vehicle (DMSO), flumazenil, or known CYP450 inducers exhibited normal hepatocyte morphology. No morphological changes were noted in hepatocytes treated with up to 40 μM FFA or 50 μM nFFA for 3 consecutive days. Treatment with up to 40 μM FFA or 50 μM nFFA caused little LDH release (≤8.5% or ≤13.8% positive control, respectively) in all three hepatocyte cultures.

**FIGURE 2:**
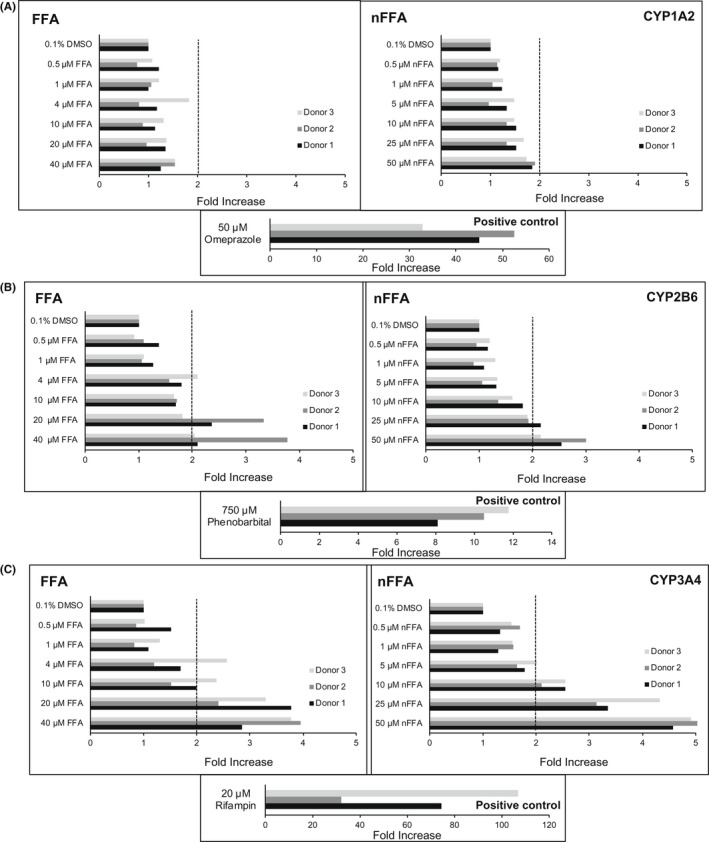
Effects of various concentrations of fenfluramine (FFA), norfenfluramine (nFFA), or control on CYP1A2, CYP2B6, and CYP3A4 mRNA levels. Primary human hepatocyte cultures from three donors were incubated for 72 h with medium containing 0−40 µM FFA, 0−50 µM nFFA, or positive and negative controls. Levels of CYP1A2 mRNA (A), CYP2B6 mRNA (B), and CYP3A4 mRNA (C) were measured by quantitative polymerase chain reaction and were normalized first to the levels of glyceraldehyde 3‐phosphate dehydrogenase mRNA, and then to the levels of corresponding CYP450 mRNAs in vehicle control cultures, as described in *Materials and Methods*. Hashed lines indicate 2‐fold induction threshold

Cultured human hepatocytes were treated with FFA or nFFA to evaluate effects of both drugs on induction of CYP1A2, CYP2B6, and CYP3A4 mRNA expression (Table 2; Figure 2). The positive control inducers caused expected increases in CYP mRNA expression in cultures of human hepatocytes. Omeprazole (50 μM), phenobarbital (750 μM), and rifampin (20 μM) caused increases ranging from 32.7‐ to 52.5‐fold, from 8.12‐ to 11.8‐fold, and from 32.0‐ to 107‐fold in CYP1A2, CYP2B6, and CYP3A4 mRNA levels, respectively (Figure 2). In all three hepatocyte cultures, both FFA (up to 40 μM) and nFFA (50 μM) had little or no effect (<2‐fold change and <20% as effective as the positive control, omeprazole) on CYP1A2 mRNA levels. FFA and nFFA changed CYP1A2 mRNA levels from 1.24− to 1.54‐fold and from 1.74− to 1.92‐fold, respectively (Table 2). These values, according to the fold‐change method, indicate lack of potential to induce the enzyme in vivo.^15^ No further estimates were conducted for CYP1A2.

**TABLE 2:** Evaluation of Fenfluramine (FFA) and norfenfluramine (nFFA) potential to induce CYP1A2, CYP2B6, or CYP3A4 mRNA in vivo

		FFA			nFFA		
		HC10‐10	HC10‐8	HC7‐8	HC10‐10	HC10‐8	HC7‐8
CYP1A2							
Fold‐change method	Fold change^a^	1.24	1.54	1.54	1.86	1.92	1.74
	% of control^b^	0.55	1.04	1.72	1.96	1.79	2.35
	Potential to induce	No	No	No	No	No	No
CYP2B6							
Fold‐change method	Fold change	2.09	3.77	2.05	2.54	3.01	2.15
	% of control	15.3	29.10	9.66	21.60	21.20	10.60
	Potential to induce	Yes	Yes	Yes	Yes	Yes	Yes
Basic kinetic model	E_max_ (fold)^c^	1.21 ± 0.21	2.80 ± 0.20	0.94 ± 0.10	1.36 ± 0.11	2.08 ± 0.197	1.14 ± 0.083
	EC_50_ (µM)	4.61 ± 3.3	13.6 ± 1.5	8.32 ± 2.1	8.81 ± 1.6	26.4 ± 2.9	10.8 ± 2.3
	R_3_ ^d^	0.76	0.77	0.87	0.88	0.93	0.92
	Potential to induce	Yes	Yes	No	No	No	No
Static mechanistic model	AUCR^d^	0.92	0.93	0.96	0.99	0.99	0.99
	Potential to induce	No	No	No	No	No	No
CYP3A4							
Fold‐change method	Fold change	2.86	3.95	3.77	4.57	5.75	4.91
	% of control	2.53	9.50	2.60	4.85	15.30	3.68
	Potential to induce	Yes	Yes	Yes	Yes	Yes	Yes
Basic kinetic model	E_max_ (fold)	2.30 ± 0.43	3.02 ± 0.25	2.69 ± 0.52	3.61 ± 0.40	6.95 ± 1.0	3.92 ± 0.048
	EC_50_ (µM)	9.05 ± 3.6	20.4 ± 1.9	8.53 ± 4.3	17.1 ± 3.9	37.8 ± 5.6	12.9 ± 0.38
	R_3_	0.74	0.82	0.70	0.84	0.86	0.79
	Potential to induce	Yes	No	Yes	No	No	Yes
Static mechanistic model	AUCR	0.92	0.95	0.90	0.96	0.97	0.95
		No	No	No	No	No	No

Purple: data relating to the basic kinetic model. Blue: data relating to the static mechanistic model.

^a^ Fold‐change in mRNA concentration at highest dose tested (40 µM fenfluramine and 50 µM norfenfluramine).

^b^ Percent of positive control induction of mRNA.

^c^ E_max_ and EC_50_ and respective standard errors were calculated from the plot of drug concentrations vs fold‐change −1 values of drug effect with Sigmoid, 3‐paramether equation (SigmaPlot).

^d^ Calculated as per FDA Guidance (2020).

Induction of CYP2B6 mRNA expression ranged from 2.05‐fold to 3.77‐fold above solvent control for FFA, and from 2.15‐fold to 3.01‐fold for nFFA (Table 2; Figure 2), both of which are above the 2‐fold threshold set by FDA for potentially clinically relevant enzyme induction.^15^ Fold‐increases in CYP3A4 mRNA levels caused by FFA and nFFA ranged from 2.86‐fold to 3.95‐fold for FFA and from 4.57‐fold to 5.75‐fold for nFFA, which are above the FDA‐specified threshold (Table 2; Figure 2).

To further investigate the potential for FFA and nFFA to induce CYP2B6 and CYP3A4 clinically, basic kinetic and static mechanistic models were applied. E_max_ and EC_50_ parameters for effects of FFA and nFFA on the fold‐increase in CYP2B6 and CYP3A4 mRNA were estimated from sigmoidal three‐parameter equations. The parameters were applied to calculate R_3_ and AUCR values for the basic kinetic and static mechanistic models, respectively (Table 2; Supplemental Table 2).^15^ The R_3_ value predicted CYP2B6 induction by FFA in two of three hepatocyte cultures but did not predict CYP2B6 induction by nFFA (Table 2). The basic kinetic model predicted CYP3A4 induction by FFA in two hepatocyte cultures, and by nFFA in one hepatocyte culture. With the static mechanistic model, neither FFA nor nFFA was predicted to induce CYP2B6 or CYP3A4 enzymes in vivo (Table 2).

### 3.4 Transporter inhibition

**FIGURE 3:**
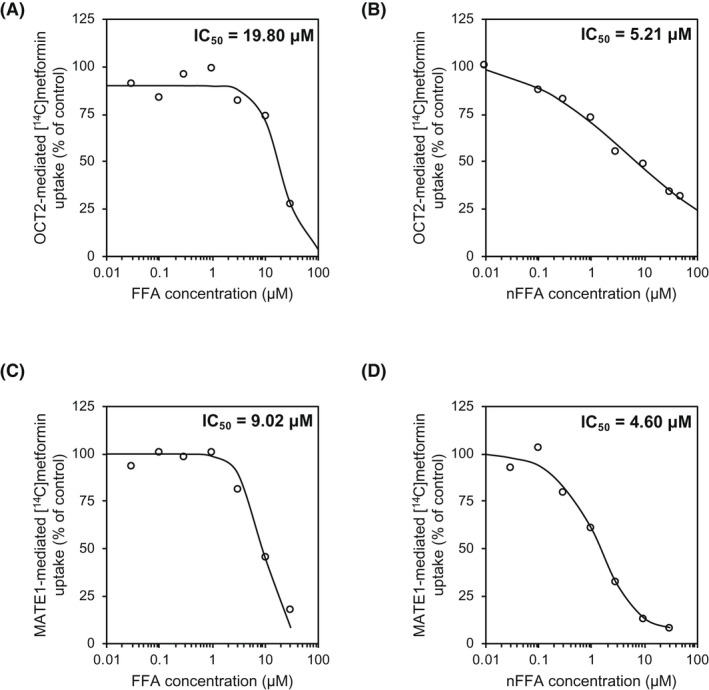
Inhibition of OCT2 and MATE1 drug transporters by fenfluramine (FFA) and norfenfluramine (nFFA). Accumulation of ^14^C‐metformin uptake into HEK‐OCT2 cells in the presence of (A) FFA or (B) nFFA. Accumulation of ^14^C‐metformin into HEK‐MATE1 cells in the presence of (C) FFA or (D) nFFA

The IC_50_ values characterizing inhibition of P‐gp, BCRP, OATP1B1, OATP1B3, OAT1, OAT3, OCT2, MATE1, and MATE2‐K transporters by FFA and nFFA, as well as the FDA recommended criteria for evaluation of drug transporter inhibition, namely, the formulae for R values and their cutoffs, are presented in Table 3.^15^ Estimated IC_50_ values were above the highest concentrations of FFA and nFFA examined for all transporters, with the exception of OCT2 and MATE1. FFA inhibited OCT2 and MATE1 transporters, with IC_50_ values of 19.8 and 9.0 µM, respectively (Figure 3A, B). nFFA inhibited OCT2 and MATE1 transporters, with IC_50_ values of 5.2 and 4.6 µM, respectively (Figure 3C, D). The potential of FFA and nFFA to inhibit drug transporters in vivo was evaluated by calculating the R values and comparing them with their predetermined cutoff values. Calculated R values for both FFA and nFFA for all drug transporters examined were below their respective cutoff values, indicating lack of potential to inhibit the transporters in vivo.

**TABLE 3:** Fenfluramine (FFA) and norfenfluramine (nFFA) potential to inhibit drug transporters—definitions and values of R

Transporter	IC_50_ ^a^ (µM)		Potential to inhibit transporter	R value	
	FFA	nFFA	(R)	FFA	nFFA
P‐gp	>100	>50	I_gut_/IC_50_ ≥ 10	<2.2511	NA
	>100	>50	C_max_/IC_50_ ≥ 0.1	NA	<0.0037
BCRP	>100	>50	I_gut_/IC_50_ ≥ 10	<2.2511	NA
	>100	>50	C_max_/IC_50_ ≥ 0.1	NA	<0.0037
OATP1B1	>300	>50	1+(f_u,p_ x I_in,max_)/IC_50_ ≥ 1.1	<1.0012	<1.0017
OATP1B3	>300	>50	1+(f_u,p_ x I_in,max_)/IC_50_ ≥ 1.1	<1.0012	<1.0017
OAT1	>30	>50	I_max,u_/IC_50_ ≥ 0.1	<0.0054	<0.0019
OAT3	>30	>50	I_max,u_/IC_50_ ≥ 0.1	<0.0054	<0.0019
OCT2	19.8	5.2	I_max,u_/IC_50_ ≥ 0.1	0.0082	0.0180
MATE1	9.0	4.6	I_max,u_/IC_50_ ≥ 0.1	0.0181	0.0204
MATE2‐K	>30	>50	I_max,u_/IC_50_ ≥ 0.1	<0.0054	<0.0019

Note

Abbreviation: NA, not applicable.

^a^ IC_50_ is calculated as described in *Materials and Methods*. See Supplemental Table 1 for transcellular transporter assay conditions.

## 4 DISCUSSION

This study evaluated FFA and its major metabolite nFFA DDI perpetrator potential in vitro and utilized modeling approaches to help translate in vitro observations into in vivo predictions of potential clinical DDIs. FFA and nFFA were found to inhibit CYP2D6 in vitro. No noteworthy inhibition of CYP1A2, CYP2B6, CYP2C8, CYP2C9, CYP2C19, or CYP3A4/5 was observed in the presence of FFA or nFFA. In the mRNA analysis, both compounds were found to induce CYP2B6 and CYP3A4. Neither compound significantly induced CYP1A2. Neither compound significantly inhibited BCRP, P‐gp, OAT1, OAT3, OCT2, MATE1, or MATE2‐K. Further evaluation with static mechanistic models^15^ predicted that FFA and nFFA were unlikely to have substantial perpetrator potential at clinically relevant doses, either by inducing CYP450s or by inhibiting transporters or CYP450 enzymes.

FFA undergoes first‐pass metabolism in the liver.^12^ Previous evidence in the literature corroborates the findings of CYP2D6 inhibition at high micromolar IC_50_ values by FFA. von Moltke et al.^35^ reported IC_50_ values of 15.1 µM for inhibition of CYP2D6‐dependent dextromethorphan O‐demethylation by both D‐ and L‐isomers isolated from the FFA racemic mixture.^35^ The IC_50_ reported by these authors is closer to our results for nFFA than for FFA. The reason for this difference is unclear but could involve subtle differences in the racemic mixture of FFA and the isolated isomers. These authors also reported weak or negligible inhibitor potential of D‐ or L‐FFA for CYP1A2, CYP2C9, and CYP3A, in accordance with our results.^35^

Of ASMs commonly used in DS and/or LGS regimens,^36^, ^37^ none are extensively metabolized by CYP2D6.^7^, ^38^ Stiripentol, for example, is metabolized by CYP1A2, CYP2C19, and CYP3A4, and clobazam is metabolized by CYP3A4 and CYP2C19.^7^, ^38^ Valproate, one of the most commonly prescribed ASMs for DS, is metabolized by CYP2C9, CYP2A6, and CYP2B6, but is not extensively metabolized by CYP2D6.^39^, ^40^

Time‐dependent CYP450 modulation may result in an underprediction of DDI potential.^41^, ^42^ However, neither FFA nor nFFA was a time‐dependent (i.e., NADPH‐independent) nor a metabolism‐dependent (i.e., both time‐ and NADPH‐dependent) inhibitor of any CYP450 enzyme tested in this study according to the standards specified in the FDA 2020 Guidance for Industry.^15^ For CYP2D6, a 30‐min preincubation with NADPH‐fortified human liver microsomes and escalating FFA doses actually increased the IC_50_ from 4.5 μM up to 10 μM, suggesting a slight decrease of in vitro inhibitory potential after prolonged CYP2D6 activation. Conversely, such an increase was not observed with nFFA, where IC_50_ increased only marginally after 30‐min incubation with NADPH when nFFA was the inhibitor. Taken together with the results of the static mechanistic model demonstrating no potential for clinical inhibition, this lack of time‐ or co‐factor‐dependent inhibition of CYP2D6 supports that FFA and nFFA are unlikely to substantially inhibit CYP2D6 enzyme activity.

The slight CYP3A4 and CYP2B6 induction observed in our study is unlikely to be clinically relevant per the static mechanistic model predictions. CYP2B6 does not appear to have a high f_m_ for any of the commonly used ASMs in LGS or DS.^7^ CYP3A4 metabolizes numerous xenobiotics including ASMs (e.g., stiripentol, perampanel, felbamate, clobazam, ethosuximide),^7^ and CYP3A4 induction increases clearance of many xenobiotics.^42^, ^43^ However, the R_3_ calculations of intrinsic clearance in our study for all evaluable samples are outside established thresholds for significant DDI potential (>0.8).^15^, ^44^, ^45^

Our in vitro results suggest that FFA is unlikely to affect drug distribution and/or elimination by inhibiting the major drug transporters.^46^ The R values were below the FDA‐specified threshold for transporter inhibition of OATP1B1 and OATP1B3 hepatic uptake transporters or OAT1, OAT3, OCT, or MATE2‐K renal transporters (Table 3).^15^ FFA and nFFA inhibition of renal clearance transporters OCT2 and MATE1^47^ was greater than that observed for the other transporters investigated (Table 3). However, the observed inhibition was not predicted to be clinically meaningful. R values for OCT2 were below FDA prespecified thresholds of ≥0.1; values for FFA and nFFA inhibition of MATE1 were <0.0181 and <0.0204, respectively, corresponding to less than the FDA‐specified threshold of ≥0.1. At clinically relevant doses, neither FFA nor nFFA has significant potential to inhibit renal or hepatic drug transporters.

Our in vitro results align with clinical reports suggesting that FFA is unlikely to have sufficient perpetrator potential when used in combination ASM regimens, although DDI have been reported. A recent clinical study investigated the impact of FFA coadministration on plasma levels of stiripentol, valproate, and clobazam—a common combination regimen prescribed for treating DS.^48^ C_max_ values of valproate, stiripentol, and clobazam, all highly protein‐bound drugs,^49^, ^50^ were not significantly affected by adding FFA.^48^ In a separate study with single‐dose FFA added to steady‐state cannabidiol, FFA did not significantly affect the pharmacokinetics of cannabidiol.^48^ Based on the current study, this result is expected, given that cannabidiol is metabolized by CYP2C19 and CYP3A4,^51^ neither of which was substantially inhibited or induced by FFA or nFFA in vitro. These clinical results support our in vitro observations that FFA is unlikely to substantially induce or inhibit any CYP450 enzyme at clinically relevant doses used in ASM regimens for LGS and DS.

The primary strength of this study is its rigorous, systematic approach to investigating perpetrator potential of FFA. Previous studies published when FFA was marketed as an anorectic agent were targeted investigations of specific CYP enzymes. By adhering to FDA and EMA Guidance to Industry, we report the most comprehensive, systematic report of FFA and nFFA perpetrator potential published to date.

FFA is a racemic mixture of two enantiomers. Although prior publications have evaluated the enantiomers separately, the clinically used FFA is administered as a racemate and therefore the experimental studies were conducted with the racemic form.

This study has some limitations. It should be noted that pharmacogenetic variants cause affected individuals to be classified as poor, extensive, or ultra‐metabolizers of CYP2D6 substrates, as well as CYP2C9, CYP2C19, and CYP3A4 substrates,^52^ although data suggest that polymorphism of any individual CYP450 is unlikely to affect FFA pharmacokinetics (Zogenix, data on file). It is unknown how interindividual differences among pharmacogenetic variants of CYP450s and drug transporters could affect local and systemic drug concentrations of FFA.

In conclusion, this study provides a comprehensive examination of the impact of FFA and nFFA on clinically relevant CYP450s and transporter proteins. The in vitro DDI data suggest limited potential for FFA to have significant perpetrator activity in multidrug ASM regimens.

## DISCLOSURE

P.M. and B.B. are employees of, and own stock in, Zogenix, Inc., S.S. is a consultant for Zogenix, Inc., B.W.O., M.C., and P.B.L. are consultants for Zogenix, Inc., and are employees of Sekisui XenoTech, LLC.

## ETHICAL STATEMENT

This study is exempt from ethics approval.

## AUTHOR CONTRIBUTION

Participated in research design: BWO, Conducted experiments: Sekisui XenoTech, LLC, Contributed new reagents or analytical tools: N/A, Performed data analysis: MC, PLB, Wrote or contributed to the writing of the manuscript: PM, SS, BB, MC, PLB, BWO.

## Supporting information

Table S1‐S3

Supplementary Material

## Data Availability

Zogenix is in the process of establishing a data sharing policy. Written requests for data by legitimate investigators/researchers/clinicians may be submitted to Zogenix, Inc. These requests will be considered on a case‐by‐case basis and reviewed for appropriateness.

## References

[1] Andrade R , Barnes NM , Baxter G , et al. Hydroxytryptamine receptors (version 2019.4) in the IUPHAR/BPS guide to pharmacology database. IUPHAR/BPS Guide Pharmacol CITE. 2019;2019(4):1‐31. doi:10.2218/gtopdb/F1/2019.4

[2] Lagae L , Sullivan J , Knupp K , et al. Fenfluramine hydrochloride for the treatment of seizures in Dravet syndrome: a randomised, double‐blind, placebo‐controlled trial. Lancet. 2019;394:2243‐2254. 31862249 10.1016/S0140-6736(19)32500-0

[3] Nabbout R , Mistry A , Zuberi S , et al. Fenfluramine for treatment‐resistant seizures in patients with Dravet syndrome receiving stiripentol‐inclusive regimens: a randomized clinical trial. JAMA Neurol. 2020;77:300‐308. 31790543 10.1001/jamaneurol.2019.4113 PMC6902175

[4] Aras LM , Isla J , Mingorance‐Le Meur A . The European patient with Dravet syndrome: results from a parent‐reported survey on antiepileptic drug use in the European population with Dravet syndrome. Epilepsy Behav. 2015;44:104‐109. 25666511 10.1016/j.yebeh.2014.12.028

[5] Devinsky O , Patel AD , Cross JH , et al. Effect of cannabidiol on drop seizures in the Lennox‐Gastaut syndrome. N Engl J Med. 2018;378:1888‐1897. 29768152 10.1056/NEJMoa1714631

[6] Lin JH . CYP induction‐mediated drug interactions: in vitro assessment and clinical implications. Pharm Res. 2006;23:1089‐1116. 16718615 10.1007/s11095-006-0277-7

[7] Zaccara G , Perucca E . Interactions between antiepileptic drugs, and between antiepileptic drugs and other drugs. Epileptic Disord. 2014;16:409‐431. 25515681 10.1684/epd.2014.0714

[8] Jahngir MU , Ahmad MQ , Jahangir M . Lennox‐gastaut syndrome: in a nutshell. Cureus. 2018;10:e3134. 30410820 10.7759/cureus.3134 PMC6207167

[9] Wirrell EC , Laux L , Donner E , et al. Optimizing the diagnosis and management of Dravet syndrome: recommendations from a North American consensus panel. Pediatr Neurol. 2017;68:18‐34. 28284397 10.1016/j.pediatrneurol.2017.01.025

[10] Devinsky O , King L , Schwartz D , Conway E , Price D . Effect of fenfluramine on convulsive seizures in CDKL5 deficiency disorder. Epilepsia. 2021;62:e98‐e102. 33979451 10.1111/epi.16923 PMC8360137

[11] Devinsky O , Verducci C , Thiele EA , et al. Open‐label use of highly purified CBD (Epidiolex®) in patients with CDKL5 deficiency disorder and Aicardi, Dup15q, and Doose syndromes. Epilepsy Behav. 2018;86:131‐137. 30006259 10.1016/j.yebeh.2018.05.013

[12] Bever KA , Perry PJ . Dexfenfluramine hydrochloride: an anorexigenic agent. Am J Health Syst Pharm. 1997;54:2059‐2072. 9377205 10.1093/ajhp/54.18.2059

[13] Marchant NC , Breen MA , Wallace D , et al. Comparative biodisposition and metabolism of ^14^C‐(+/‐)‐fenfluramine in mouse, rat, dog and man. Xenobiotica. 1992;22:1251‐1266. 1492418 10.3109/00498259209053154

[14] Zientek MA , Youdim K . Reaction phenotyping: advances in the experimental strategies used to characterize the contribution of drug‐metabolizing enzymes. Drug Metab Dispos. 2015;43:163‐181. 25297949 10.1124/dmd.114.058750

[15] US Food and Drug Administration . In vitro drug interaction studies—cytochrome P450 enzyme‐ and transporter‐mediated drug interactions. Guidance for industry, 2020.

[16] Giraud C , Treluyer JM , Rey E , et al. In vitro and in vivo inhibitory effect of stiripentol on clobazam metabolism. Drug Metab Dispos. 2006;34:608‐611. 16415114 10.1124/dmd.105.007237

[17] Morrison G , Crockett J , Blakey G , Sommerville K . A phase 1, open‐label, pharmacokinetic trial to investigate possible drug‐drug interactions between clobazam, stiripentol, or valproate and cannabidiol in healthy subjects. Clin Pharmacol Drug Dev. 2019;8:1009‐1031. 30791225 10.1002/cpdd.665 PMC6899822

[18] Di L . Reaction phenotyping to assess victim drug‐drug interaction risks. Expert Opin Drug Discov. 2017;12:1105‐1115. 28820269 10.1080/17460441.2017.1367280

[19] Ogilvie BW , Usuki E , Yerino P , Parkinson A . In vitro approaches for studying the inhibition of drug‐metabolizing enzymes responsible for the metabolism of drugs (reaction phenotyping) with emphasis on cytochrome P450. In: Rodrigues AD , ed. Drug‐drug interactions. Informa Healthcare USA; 2008:231‐358.

[20] Parkinson A , Ogilvie BW , Buckley DB , Kazmi F , Czerwinski M , Parkinson O . Biotransformation of xenobiotics. In: Klaassen CD , ed. Casarett & Doull’s toxicology, the basic science of poisons. McGraw‐Hill Companies Inc; 2013:185‐376.

[21] Hillgren KM , Keppler D , Zur AA , et al. Emerging transporters of clinical importance: an update from the international transporter consortium. Clin Pharmacol Ther. 2013;94:52‐63. 23588305 10.1038/clpt.2013.74

[22] Beckett AH , Brookes LG . The absorption and urinary excretion in man of fenfluramine and its main metabolite. J Pharm Pharmacol. 1967;19(suppl):42S‐49S. 4383855

[23] Martin P , Czerwiński M , Limaye PB , et al. In vitro evaluation of fenfluramine and norfenfluramine as victims of drug interactions. Pharmacol Res Perspect. 2022;10:e00958. doi:10.1002/prp2.959 PMC9124820 35599345

[24] Haupt LJ , Kazmi F , Ogilvie BW , et al. The reliability of estimating κ_i_ values for direct, reversible inhibition of cytochrome P450 enzymes from corresponding IC_50_ values: a retrospective analysis of 343 experiments. Drug Metab Dispos. 2015;43:1744‐1750. 26354951 10.1124/dmd.115.066597

[25] Parkinson A , Mudra DR , Johnson C , Dwyer A , Carroll KM . The effects of gender, age, ethnicity, and liver cirrhosis on cytochrome *P*450 enzyme activity in human liver microsomes and inducibility in cultured human hepatocytes. Toxicol Appl Pharmacol. 2004;199:193‐209. 15364537 10.1016/j.taap.2004.01.010

[26] Pearce RE , McIntyre CJ , Madan A , et al. Effects of freezing, thawing, and storing human liver microsomes on cytochrome P450 activity. Arch Biochem Biophys. 1996;331:145‐169. 8660694 10.1006/abbi.1996.0294

[27] Paris BL , Ogilvie BW , Scheinkoenig JA , Ndikum‐Moffor F , Gibson R , Parkinson A . In vitro inhibition and induction of human liver cytochrome p450 enzymes by milnacipran. Drug Metab Dispos. 2009;37:2045‐2054. 19608694 10.1124/dmd.109.028274

[28] Vermeer LMM , Isringhausen CD , Ogilvie BW , Buckley DB . Evaluation of ketoconazole and its alternative clinical CYP3A4/5 inhibitors as inhibitors of drug transporters: the in vitro effects of ketoconazole, ritonavir, clarithromycin, and itraconazole on 13 clinically‐relevant drug transporters. Drug Metab Dispos. 2016;44:453‐459. 26668209 10.1124/dmd.115.067744

[29] Izumi S , Nozaki Y , Komori T , et al. Substrate‐dependent inhibition of organic anion transporting polypeptide 1B1: comparative analysis with prototypical probe substrates estradiol‐17 β‐glucuronide, estrone‐3‐sulfate, and sulfobromophthalein. Drug Metab Dispos. 2013;41:1859‐1866. 23920221 10.1124/dmd.113.052290

[30] Harding SD , Sharman JL , Faccenda E , et al. The IUPHAR/BPS Guide to PHARMACOLOGY in 2019: updates and expansion to encompass the new guide to IMMUNOPHARMACOLOGY. Nucleic Acids Res. 2018;46:D1091‐1106. doi:10.1093/nar/gkx1121 29149325 PMC5753190

[31] Alexander SPH , Christopoulos A , Davenport AP , et al. The concise guide to pharmacology 2021/22: G protein‐coupled receptors. Br J Pharmacol. 2021;178:S27‐S156. doi:10.1111/bph.15538 34529832

[32] Alexander SPH , Fabbro D , Kelly E , et al. The concise guide to pharmacology 2021/22: Enzymes. Br J Pharmacol. 2021;178:S313‐S411. doi:10.1111/bph.15542 34529828

[33] Alexander SPH , Kelly E , Mathie A , et al. The concise guide to pharmacology 2021/22: transporters. Br J Pharmacol. 2021;178:S412‐S513. doi:10.1111/bph.15543 34529826

[34] Holford NH . Clinical pharmacokinetics and pharmacodynamics of warfarin. Understanding the dose‐effect relationship. Clin Pharmacokinet. 1986;11:483‐504. 3542339 10.2165/00003088-198611060-00005

[35] von Moltke LL , Greenblatt DJ , Ciraulo DA , et al. Appetite suppressant drugs as inhibitors of human cytochromes P450: in vitro inhibition of P450–2D6 by D‐ and L‐fenfluramine, but not phentermine. J Clin Psychopharmacol. 1998;18:338‐341. 9690701 10.1097/00004714-199808000-00015

[36] McTague A , Cross JH . Treatment of epileptic encephalopathies. CNS Drugs. 2013;27:175‐184. 23397290 10.1007/s40263-013-0041-6

[37] Wallace A , Wirrell E , Kenney‐Jung DL . Pharmacotherapy for Dravet syndrome. Pediatric Drugs. 2016;18:197‐208. 26966048 10.1007/s40272-016-0171-7

[38] Johannessen Landmark C , Patsalos PN . Drug interactions involving the new second‐ and third‐generation antiepileptic drugs. Expert Rev Neurother. 2010;10:119‐140. 20021326 10.1586/ern.09.136

[39] Ghodke‐Puranik Y , Thorn CF , Lamba JK , et al. Valproic acid pathway: pharmacokinetics and pharmacodynamics. Pharmacogenet Genomics. 2013;23:236‐241. 23407051 10.1097/FPC.0b013e32835ea0b2 PMC3696515

[40] Kiang TK , Ho PC , Anari MR , Tong V , Abbott FS , Chang TK . Contribution of CYP2C9, CYP2A6, and CYP2B6 to valproic acid metabolism in hepatic microsomes from individuals with the CYP2C9*1/*1 genotype. Toxicol Sci. 2006;94:261‐271. 16945988 10.1093/toxsci/kfl096

[41] Fahmi OA , Maurer TS , Kish M , Cardenas E , Boldt S , Nettleton D . A combined model for predicting CYP3A4 clinical net drug‐drug interaction based on CYP3A4 inhibition, inactivation, and induction determined in vitro. Drug Metab Dispos. 2008;36:1698‐1708. 18490437 10.1124/dmd.107.018663

[42] Fahmi OA , Ripp SL . Evaluation of models for predicting drug‐drug interactions due to induction. Expert Opin Drug Metab Toxicol. 2010;6:1399‐1416. 20955108 10.1517/17425255.2010.516251

[43] Fahmi OA , Boldt S , Kish M , Obach RS , Tremaine LM . Prediction of drug‐drug interactions from in vitro induction data: application of the relative induction score approach using cryopreserved human hepatocytes. Drug Metab Dispos. 2008;36:1971‐1974. 18519654 10.1124/dmd.108.021907

[44] Tachibana T , Kato M , Watanabe T , Mitsui T , Sugiyama Y . Method for predicting the risk of drug‐drug interactions involving inhibition of intestinal CYP3A4 and P‐glycoprotein. Xenobiotica. 2009;39:430‐443. 19480549 10.1080/00498250902846252

[45] Vieira ML , Kirby B , Ragueneau‐Majlessi I , et al. Evaluation of various static in vitro‐in vivo extrapolation models for risk assessment of the CYP3A inhibition potential of an investigational drug. Clin Pharmacol Ther. 2014;95:189‐198. 24048277 10.1038/clpt.2013.187

[46] Giacomini KM , Huang SM , Tweedie DJ , et al. Membrane transporters in drug development. Nat Rev Drug Discovery. 2010;9:215‐236. 20190787 10.1038/nrd3028 PMC3326076

[47] Yin J , Duan H , Wang J . Impact of substrate‐dependent inhibition on renal organic cation transporters hOCT2 and hMATE1/2‐K‐mediated drug transport and intracellular accumulation. J Pharmacol Exp Ther. 2016;359:401‐410. 27758931 10.1124/jpet.116.236158 PMC5118648

[48] Boyd B , Smith S , Gammaitoni A , Galer BS , Farfel GM . A phase I, randomized, open‐label, single‐dose, 3‐period crossover study to evaluate the drug‐drug interaction between ZX008 (fenfluramine HCl oral solution) and a regimen of stiripentol, clobazam, and valproate in healthy subjects. Int J Clin Pharmacol Ther. 2019;57:11‐19. 30336805 10.5414/CP203276 PMC6298132

[49] Biocodex . DIACOMIT (stiripentol) capsules, for oral use. DIACOMIT (stiripentol) powder, for oral suspension [prescribing information]. Biocodex. 2018.

[50] Patsalos PN , Zugman M , Lake C , James A , Ratnaraj N , Sander JW . Serum protein binding of 25 antiepileptic drugs in a routine clinical setting: a comparison of free non‐protein‐bound concentrations. Epilepsia. 2017;58:1234‐1243. 28542801 10.1111/epi.13802

[51] Stout SM , Cimino NM . Exogenous cannabinoids as substrates, inhibitors, and inducers of human drug metabolizing enzymes: a systematic review. Drug Metab Rev. 2014;46:86‐95. 24160757 10.3109/03602532.2013.849268

[52] Lopez‐Garcia MA , Feria‐Romero IA , Serrano H , et al. Influence of genetic variants of *CYP2D6*, *CYP2C9*, *CYP2C19* and *CYP3A4* on antiepileptic drug metabolism in pediatric patients with refractory epilepsy. Pharmacol Rep. 2017;69:504‐511. 10.1016/j.pharep.2017.01.007 28343093

