# Supplementary material for: In vitro evaluation suggests fenfluramine and norfenfluramine are unlikely to act as perpetrators of drug interactions
Source: Pharmacol Res Perspect. 2022 May 22;10(3):e00959. doi: 10.1002/prp2.959 (PMC9124818; doi:10.1002/prp2.959)
Supplement: Supplementary file 1 — Table S1‐S3 [file PRP2-10-e00959-s001.docx]

Supplemental Table 1

Transcellular transporter assays

| Transporter  (cell line) | Probe Substrate  (concentration) | Concentration Range (µM) | |
| --- | --- | --- | --- |
|  |  | FFA | nFFA |
| P-gp (Caco-2) | Digoxin (10 µM) | 0.3–100 | 0.3–50 |
| BCRP (MDCKII) | Prazosin (1 µM) | 0.3–100 | 0.3–50 |
| OATP1B1 (HEK293) | [^3^H]-Estradiol-17β-glucuronide (50 nM) [^3^H]-Estradiol-17β-glucuronide (50 nM) | 0.3–300 | 0.1–50 |
| OATP1B3 (HEK293) |  | 0.3–300 | 0.1–50 |
| OAT1 (HEK293) | [^3^H]-p-Aminohippurate (1 μM) | 0.03–30 | 0.1–50 |
| OAT3 (HEK293) | [^3^H]-Estrone-3-sulfate (50 nM) | 0.03–30 | 0.1–50 |
| OCT2 (HEK293) | [^14^C]-Metformin (10 μM) [^14^C]-Metformin (10 μM)  [^14^C]-Metformin (10 μM) | 0.03–30 | 0.1–50 |
| MATE1 (HEK293) |  | 0.03–30 | 0.1–50 |
| MATE2-K (HEK293) |  | 0.03–30 | 0.1–50 |

Supplemental Table 2

Calculation of the potential for FFA and nFFA to inhibit CYP2D6: parameters for mechanistic static model

| Parameter | FFA | nFFA | Source |
| --- | --- | --- | --- |
| Molecular weight (MW) | 231 | 203 | Chemical structure |
| Fraction unbound (fu) | 55.2% | 50.3% | Current study |
| IC_50_ (μM) | 4.7 | 16 | Current study |
| Ki, u (μM) | 2.17 | 7.86 | IC_50_ divided by 2, assumes  [S] = Km; also adjusted for estimated unbound fraction (fu) in microsomes |
| Blood/plasma ratio (Rb) | 1.11 | NA | Current study (see *Methods*) |
| Dose (mg) | 13a | NA | Maximum dose 26 mg/day,  13 mg/dose BID fenfluramine only, expressed as fenfluramine free base |
| C_max, total_ (ng/mL) | 68.0 | 37.8 | Modeled (PopPK) |
| C_max, unbound_ (μM) | 0.162 | 0.094 | Calculated as C_max_, total (ng/mL)/MW * fu |
| Q_en_ (L/h) | 18 | 18 | From 2020 FDA guidance, liver blood flow (Yang 2017a) |
| Q_h_ (L/h) | 97 | 97 | From 2020 FDA guidance, liver blood flow (Yang 2017b) |
| k_a_ (h-1) | 0.69 | NA | Modeled (PopPK) |
| Fraction absorbed (F_a_) | 1 | NA | Most conservative estimate; yields highest possible portal concentration |
| Fraction that reaches the portal vein (F_g_) | 1 | NA | Assumes no intestinal extraction (little CYP2D6 in the intestine) |
| Portal inlet [I]_h_ (μM) | 0.362 | 0.094 | Calculated as (I_max_ + (F_a_ × F_g_ × k_a_ × molar dose) / Q_h_) / Rb |
| A_h_ (guidance term) | 0.867 | 0.988 | Calculated from 2020 FDA guidance |
| B_h_ (guidance term) | 1 | 1 | Calculated from 2020 FDA guidance (TDI set to 1 because there is no TDI based on the current study) |
| F_m_ victim | 1 | 1 | Assumes 100% metabolism by target CYP450 susceptible to inhibition (most conservative value) |
| AUCR (R1) | 1.15 | 1.01 | Calculated from 2020 FDA guidance |

A_h_, B_h_, and AUCR calculated per 2020 FDA guidance (Fig. 7). Intestinal metabolism was not included because CYP2D6 does not have a significant role in intestinal metabolism.

FFA, fenfluramine; nFFA, norfenfluramine; PopPK, population pharmacokinetic model.

Supplemental Table 3

Human hepatocyte preparation: organ donor information

| XT Liver Number | Gender | Age (years) | Ethnicity | Tobacco  Use^a^ | Alcohol  Use^a^ | Plating Viability (%)^b^ |
| --- | --- | --- | --- | --- | --- | --- |
| HC10-10 | F | 56 | Caucasian | No | 3–5 drinks/week | 83 |
| HC10-8 | F | 57 | Caucasian | 3 packs/week | bottle of wine every other day | 81.7 |
| HC7-8 | M | 41 | African American | 1 PPD for 15 years | 6-pack of beer 3−4 times/year | 91 |

^a^Information provided by the Organ Procurement Organization, as reported by the next of kin.

^b^Determined by Trypan blue staining.

PPD, pack per day.
