# Supplementary material for: In vitro evaluation suggests fenfluramine and norfenfluramine are unlikely to act as perpetrators of drug interactions
Source: Pharmacol Res Perspect. 2022 May 22;10(3):e00959. doi: 10.1002/prp2.959 (PMC9124818; doi:10.1002/prp2.959)
Supplement: Supplementary file 2 — Supplementary Material [file PRP2-10-e00959-s002.docx]

**Supplemental Material**

**In Vitro Evaluation Suggests Fenfluramine and Norfenfluramine are Unlikely to Act as Perpetrators of Drug Interactions***

Parthena Martin, Maciej Czerwiński, Pallavi B. Limaye, Brian W. Ogilvie, Steven Smith, and Brooks Boyd

***Zogenix, Inc., Emeryville, CA, USA (P.M., B.B.); Consultant, Zogenix, Inc. (S.S.);
Sekisui XenoTech, LLC, Kansas City, KS, USA (M.C., P.B.L., B.W.O.)***

1. **Analytical Methods for Examination of Cytochrome P450 Inhibition**

All metabolite analyses were performed with liquid chromatography tandem mass spectrometry (LC-MS/MS) methods. The MS equipment was an API instrument (AB SCIEX) with Shimadzu high performance liquid chromatography (HPLC) pumps and autosampler systems.

| **Enzyme** | **Metabolite monitored** | **API instrument** | **HPLC column^a^** |
| --- | --- | --- | --- |
| CYP1A2 | Acetaminophen | 4500 | Waters Atlantis dC18 (5 μm, 100 mm × 2.1 mm) or Phenomenex Gemini NX C18 (3 μm, 50 mm × 2.0 mm) |
| CYP2B6 | 8-Hydroxyefavirenz | 4500 | Waters Atlantis dC18 (5 μm, 100 mm × 2.1 mm) |
| CYP2C8 | N-Desethylamodiaquine | 2000 or 4000 | Phenomenex Synergy POLAR RP 80A, (4 μm, 100 × 2.00 mm) or Waters Atlantis dC18 (5 μm, 100 mm × 2.1 mm) |
| CYP2C9 | 4′-Hydroxydiclofenac | 3000 or 4000 | Waters Atlantis dC18 (5 μm, 100 mm × 2.1 mm) |
| CYP2C19 | 4′-Hydroxymephenytoin | 5500 |  |
| CYP2D6 | Dextrorphan | 2000 |  |
| CYP3A4/5 | 1′-Hydroxymidazolam | 2000 |  |
| CYP3A4/5 | 6β-Hydroxytestosterone | 4500 | Phenomenex Gemini NX C18 (3 μm, 50 mm × 2.0 mm) |

^a^All HPLC columns were preceded by a Phenomenex Luna C-8 guard column (4.0 mm × 2.0 mm).

CYP, cytochrome P450.

Authentic metabolite standards were used, and deuterated metabolites were used as internal standards in all assays. Zero-time incubations served as blanks.

Metabolites were quantified by reference to a standard calibration curve based on back calculation of a weighted (1/x), linear, least-squares regression. The regression fit was based on the peak ratio of the analyte to an internal standard calculated from calibration standard samples, which were prepared from authentic metabolite standards. Peak areas were integrated with Analyst Instrument Control and Data Processing Software (AB SCIEX, version 1.6.1).

**Statistical Tests and Data Processing**

IC_50_ (inhibitor concentration that causes a 50% decrease in enzyme activity) data were processed with a laboratory information management system (LIMS; includes Galileo version 3.3, Thermo Fisher Scientific Inc., and reporting tool, Crystal Reports 2008, SAP). When inhibition was observed during the IC_50_ determination experiment, the data were processed for the determination of IC_50_ values by nonlinear regression and displayed on an appropriate plot. This LIMS utilizes the Levenberg-Marquardt algorithm to perform nonlinear regression fitting of the data to the following 4-parameter sigmoidal-logistic IC_50_ equation:

(Max – Min)

Y = Min +

(1 + (Conc/IC_50_)^slope^)

where Min indicates minimum value and Max indicates maximum value.

As percent of control values are utilized, the Min is set to 0 and the Max is set to 100 (or other values, as appropriate). This software has been verified for its ability to calculate an IC_50_ value only when it lies within the concentration range of inhibitors studied. Therefore, when an IC_50_ value falls outside the concentration range studied, the IC_50_ values are reported to be greater than the highest concentrations of test article evaluated.

1. **Analytical Methods for Examination of CYP Induction**

**mRNA Analysis**

mRNA assessment was conducted using a quantitative reverse transcription-polymerase chain reaction (qRT-PCR) according to the Applied Biosystems protocol. Each PCR was performed in triplicate. A Primer Mix was prepared for each Gene Expression assay. A typical Primer Mix contained TaqMan Fast Advanced Master Mix (1X), Gene Expression Assay (1X, 900 nM forward and reverse primers) and ribonuclease (RNase)-free water. The Reaction Mix was prepared by adding the Primer Mix to cDNA. A percentage of samples (no less than 10%) included NACs. (NACs are RNA samples that are not reverse-transcribed and are used to show that mRNA, not genomic DNA, is the source of a PCR’s fluorescent signal.) Reactions were analyzed on an Applied Biosystems Real Time PCR sequence detection system (AB 7900HT). The relative quantity of the target cDNA compared with that of the control cDNA (GAPDH) was determined by the ΔΔC_t_ method (Applied Biosystems User Bulletin #2). Relative quantitation measures the change in mRNA expression in a test sample relative to that in a control sample (eg, dimethyl sulfoxide [DMSO]). This method assumes that the efficiency of the target amplification and the efficiency of the endogenous control amplification are approximately equal.

**Data Processing**

**Cytotoxicity Assessment (Lactate Dehydrogenase [LDH] Release)**

Data were processed with KC4 Signature software (version 3.4, Rev 21, BioTek Instruments, Inc.) and graphed with Microsoft Excel 2003 (Microsoft Corp.). Percent LDH release was calculated based on the following equations:

ABCVM of experimental culture − ABCVM of low control

LDH release (%) = × 100

ABCVM of high control − ABCVM of low control

ABCVM = Average background − Corrected V_max_

Low control is MCM+ only.

High control is 1% Triton-X 100 in MCM+.

MCM+, modified Eagle medium Dr. Chee’s modification, supplemented; V_max_, maximum velocity of metabolism.

**mRNA by qRT-PCR**

For qRT-PCR, data were processed and graphed using a LIMS (includes Galileo version 3.3, Thermo Fisher Scientific Inc., and reporting tool, Crystal Reports 2008, SAP) and the Sequence Detection System (SDS) Software version 2.4, for Relative Quantification (Applied Biosystems). This software analyzes relative gene expression using the comparative C_t_ method (ΔΔC_t_), which relates the PCR signal of the target transcript to the PCR signal of the target in an untreated control. Both the treated sample and the untreated control signals are normalized to the endogenous control (GAPDH), for which expression is not affected by treatment and expression is constant throughout the tissue being tested. The results of this method are expressed as a fold change in expression with respect to the target transcript expression in the untreated control.

Calculations:

1. ΔC_t_ = C_t_ (target) − C_t_ (endogenous control)

2. ΔΔC_t_ = ΔC_t_ (treated sample) − ΔC_t_ (untreated control)

3. Fold change in expression = 2^−ΔΔCt^

An algorithm within the software automatically removed outliers from analysis. The statistical method used by the software is based on a modified Grubbs outlier removal (also known as the Maximum Normalized Residual Test), which permits the exclusion of a single outlier in a population consisting of as few as three replicates. However, if an apparent outlier is within 0.25 C_t_ of the mean for the associated replicate group, the software does not remove it. Outliers are considered to be wells with C_t_ values that differ significantly from associated replicate wells and typically are wells that did not amplify sufficiently, if at all.

The level of mRNA expression relative to the positive control was calculated as follows:

[(Fold change in treated sample) – 1]

Percent positive control = × 100

[(Fold change in positive control) – 1]

**EC_50_ and E_max_**

When the data allowed, EC_50_, E_max_ (maximal fold-increase in mRNA), and standard errors for each parameter for CYP induction response were calculated using SigmaPlot 12.5 based on the following equations:

Sigmoidal 3-parameter

f(x) = a/1 + exp(−(x – x_0_)/b)

where a = E_max_, b = slope, and x_0_ = EC_50_

E_max_ is defined as the maximal fold-increase in induction in vitro, and EC_50_ is defined as the concentration of inducer associated with half-maximal induction.

1. **Analytical Methods for Examination of Transporter Inhibition**

**Bidirectional permeability**

All analyses were performed with LC-MS/MS methods. The MS equipment was an API instrument (AB SCIEX) with Shimadzu HPLC pumps and autosampler systems.

| **Transporter** | **Probe substrate monitored** | **Internal standard** | **[Internal standard] (ng/mL)^a^** | **Mass spectrometer** | **ESI mode** | **HPLC column^b^** |
| --- | --- | --- | --- | --- | --- | --- |
| P-gp | Digoxin | Digoxin-d_3_ | 1000 | API 5500 | Positive | Waters Atlantis dC18  (5 μm, 100 × 2.1 mm) |
| BCRP | Prazosin | Prazosin-d_8_ | 30 |  |  | Phenomenex Gemini NX C18  (3 μm, 50 × 2.0 mm) |

^a^Value indicates the stock concentration of internal standard. This concentration is diluted 2.67-fold when added to the stopped incubation mixture.

^b^All HPLC columns were preceded by a Phenomenex Luna C-8 guard column (4 × 2.0 mm).

BCRP, breast cancer resistance protein; ESI, electrospray ionization; P-gp, permeability-glycoprotein.

Authentic standards were used, and deuterated analytes were used as internal standards. Probe substrates were quantified by back calculation of a weighted (1/x), linear, least-squares regression. The regression fit was based on analyte/internal standard peak-area ratios calculated from calibration standard samples, which were prepared from authentic standards. Peak areas were integrated with Analyst Instrument Control and Data Processing Software (AB SCIEX, version 1.6.1).

**SLC (Solute Carrier) Transport**

Samples incubated with radiolabeled probe substrates were analyzed using a liquid scintillation counter (MicroBeta^2^).

**Analytical method for the determination of fenfluramine and norfenfluramine as substrates**

For transwell assays, incubation samples (100 μL) containing fenfluramine and norfenfluramine in incubation medium were mixed with 25 μL standard blank (50:50 v/v methanol:water) and 75 μL internal standard in (50:50 v/v methanol:water). Samples expected to be higher than the standard curve range (ie, donor solutions) were diluted with incubation medium prior to being mixed with standard blank and internal standard. Standards were prepared by diluting the analyte in 50:50 v/v methanol:water at concentrations 4-fold higher than the final standard concentration and spiking into incubation medium (100 μL) and adding 75 μL internal standard in 50:50 v/v methanol:water. Fenfluramine and norfenfluramine standard curves ranged from 1 to 20000 nM and 1 to 20000 nM, respectively.

For SLC transporter assays, samples were generated by lysing cells with 50:50 v/v methanol:water containing internal standard. Standards were prepared in 50:50 v/v methanol:water containing the internal standard. Substrate solutions in incubation media were mixed with an equal volume of 50:50 v/v methanol:water containing internal standard.

**Instrumentation**

| **Instrument** | **Manufacturer** |
| --- | --- |
| Analyst Instrument Control and Data Processing software (v. 1.6.1) for collecting and integrating data | AB SCIEX |
| API 4000 mass spectrometer |  |

**HPLC columns**

| **Column** | **Manufacturer** |
| --- | --- |
| Luna C8 guard column (4.0 mm × 2.0 mm) | Phenomenex |
| Atlantis dC18 analytical column (100 × 2.1 mm, 5 μm) | Waters |

**Chromatographic parameters**

| Mobile phase A | 0.2% v/v Formic acid in water |
| --- | --- |
| Mobile phase B | 0.2% v/v Formic acid in acetonitrile |

**Fenfluramine liquid chromatography method**

| **Time (min)** | **%B** | **Flow rate (mL/min)** |
| --- | --- | --- |
| 0 | 25 | 0.6 |
| 0.50 | 25 |  |
| 1.90 | 25 |  |
| 1.95 | 25 | 0.75 |
| 2.00 | 80 | 0.6 |
| 2.10 | 95 |  |
| 2.60 | 95 |  |
| 2.65 | 25 |  |
| 3.10 | Stop |  |

At 1.95 minutes, the flow rate was increased from 0.6 to 0.75 mL/min to reduce the column re-equilibration time. The flow rate returned to 0.6 mL/min at the initiation of the next injection, or time zero.

**Norfenfluramine liquid chromatography method**

| **Time (min)** | **%B** | **Flow rate (mL/min)** |
| --- | --- | --- |
| 0 | 40 | 0.5 |
| 0.20 | 40 |  |
| 2.10 | 95 |  |
| 2.50 | 95 |  |
| 2.51 | 40 |  |
| 2.80 | Stop |  |

**Mass spectrometer parameters**

| Electrospray voltage (V) | 4500 | |
| --- | --- | --- |
| Electrospray ionization mode | Positive | |
| Mass transitions (*m/z*) | Fenfluramine | 232.0/109.0 |
|  | Norfenfluramine | 204.0/159.0 |
|  | Hydroxybupropion-d_6_ (internal standard) | 262.0/244.0 |

**In-Process Control Limits for Analytical Methods**

LC-MS/MS sample analysis and acceptance criteria are detailed in the table below. If acceptance criteria were not met, the analysis was rejected. For the calibration standards, the acceptable in-process range was 75% to 125%.

**Statistical Tests and Data Processing**

**Bidirectional Permeability**

Analyst Instrument Control and Data Processing Software (AB SCIEX, version 1.6.1) was used for probe substrate data collection and integration, which were then processed with the spreadsheet computer program Microsoft Excel 2007 (Microsoft Corp.). Calibration standards were employed to calculate concentration based on analyte/internal standard peak-area ratios using Analyst Instrument Control and Data Processing Software (AB SCIEX, version 1.6.1).

Lucifer yellow data were collected and processed with a Synergy HT Multi-Detection Microplate Reader (BioTek Instruments, Inc.).

The apparent permeability (P_app_) was calculated according to the following equation:

1

A_0_ x C_0_

dQ

dT

P_app_ = x

dQ: Transported amount of test drug in mol.

dT: Incubation time in seconds.

A_0_: Surface of porous membrane in cm^2^ (standard: 0.33).

C_0_: Initial concentration of the test drug in the donor chamber in mol/cm^3^_._

The efflux ratio (ER) for MDCKII substrate determination was calculated according to the following equation:

P_app_ B-A

P_app_ A-B

ER =

For MDCKII cells, a corrected ER was calculated according to the equation:

ER on MDCKII transporter cells

ER on MDCKII control cells

Corrected ER =

Percent of control (for MDCKII cells) was calculated with the following equation:

Corrected ER in the presence of inhibitor – 1

Corrected ER in the absence of inhibitor − 1

Percent of control (%) = × 100%

The net flux for human epithelial colorectal adenocarcinoma cells (Caco-2; P-gp) and MDCKII-BCRP inhibition determination was calculated according to the equation:

Net flux = P_app_ B-A – P_app_ A-B

The corrected net flux for MDCKII-BCRP inhibition determination was calculated according to the equation:

Corrected net flux = Net flux_transfected MDCKII_ – Net flux_control MDCKII_

Percent of control was calculated by dividing corrected net flux in the presence of inhibitor by the corrected net flux of the solvent control and multiplying by 100.

IC_50_ values were determined from the decrease in efflux ratios or net flux (percent of control) in the presence of inhibitors when inhibition exceeded 50% and calculated according to the equation:

(Max – Min)

Y = Min +

(1 + (Conc/IC_50_)^slope^)

Recovery was calculated according to the following equation:

Recovery = x 100

Q_apical_ + Q_basolateral_

Q_0_

Q_apical_ represents amount of substrate detected in the apical compartment.

Q_basolateral_ represents amount of substrate detected in the basolateral compartment.

Q_0_ represents amount of substrate detected at time zero.

The Dixon Q Test with a critical value of 5% was used to determine statistical outlying data points. The Dixon Q Test for triplicate determinations was performed with the following equation:

(x_2_ – x_1_)

(x_n_ – x_1_)

r_10_ =

x_1_: Suspected outlier.

x_n_: Replicate furthest from outlier.

x_2_: Replicate closest to outlier.

r_10_: Critical value (1-tailed 5% value = 0.941, n=3).

**SLC Transport**

Analyst Instrument Control and Data Processing Software (AB SCIEX, version 1.6.1) was used for probe substrate data collection and integration, which were then processed with the spreadsheet computer program Microsoft Excel 2007 (Microsoft Corp.). Calibration standards were employed to calculate concentration based on analyte/internal standard peak-area ratios using Analyst Instrument Control and Data Processing Software (AB SCIEX, version 1.6.1).

Liquid scintillation counter data were collected and processed with the 2450 MicroBeta^2^ Windows Workstation and then processed with the spreadsheet computer program Microsoft Excel 2007 (Microsoft Corp.).

The transporter-specific uptake of the probe substrate in cells was determined by subtracting the accumulation in the control cells from the accumulation in the transporter-expressing cells.

Percent of control was calculated by dividing transporter-specific uptake in the presence of inhibitor by the transporter-specific uptake in the solvent control and multiplying by 100.

IC_50_ values were determined from the decrease in accumulation (percent of control) in the presence of inhibitors when inhibition exceeded 50% and calculated according to the equation:

(Max – Min)

Y = Min +

(1 + (Conc/IC_50_)^slope^)

For SLC transporter substrate assays, the uptake ratio was calculated by dividing the accumulation in the transporter-expressing cells by the accumulation in control cells.

**Protein Concentration**

The concentration of protein was determined with a Synergy HT Multi-Detection Microplate Reader (BioTek Instruments, Inc.). Final protein concentrations were calculated by back calculation of an unweighted, quadratic regression with KC4 Signature software (version 3.4, rev 21, BioTek Instruments, Inc.).
